# Efficacy of Inactivated Bivalent SARS-CoV-2 Vaccines Targeting Ancestral Strain (ERAGEM), Delta, and Omicron Variants

**DOI:** 10.3390/vaccines13020169

**Published:** 2025-02-10

**Authors:** Busra Kaplan, Shaikh Terkis Islam Pavel, Muhammet Ali Uygut, Merve Tunc, Yesari Eroksuz, Ilhami Celik, Esma Eryilmaz Eren, Gulay Korukluoglu, Ates Kara, Aykut Ozdarendeli, Hazel Yetiskin

**Affiliations:** 1 Vaccine Research and Development Institute, Erciyes University, 38280 Kayseri, Türkiye; busrakaplan@erciyes.edu.tr (B.K.); pavel@erciyes.edu.tr (S.T.I.P.); mauygut@erciyes.edu.tr (M.A.U.); mervetunc368@gmail.com (M.T.); aozdarendeli@erciyes.edu.tr (A.O.); 2 Department of Pathology, Faculty of Veterinary Medicine, Fırat University, 23100 Elazig, Türkiye; yeroksuz@firat.edu.tr; 3 Department of Infectious Disease and Clinical Microbiology, University of Health Sciences, 38080 Kayseri, Türkiye; ilhami.celik@sbu.edu.tr; 4 Department of Infectious Diseases and Clinical Microbiology, Kayseri City Education and Research Hospital, 38080 Kayseri, Türkiye; esmaereneryilmaz@gmail.com; 5 Department of Clinical Microbiology, University of Health Sciences, Ankara Bilkent City Hospital, 06800 Ankara, Türkiye; 6 Pediatric Infectious Department, Faculty of Medicine, Hacettepe University Hospitals, 06230 Ankara, Türkiye; ateskara@hacettepe.edu.tr; 7 Department of Medical Microbiology, Faculty of Medicine, Erciyes University, 38280 Kayseri, Türkiye

**Keywords:** SARS-CoV-2, bivalent vaccine, inactivated vaccine, immune response, pandemic preparedness

## Abstract

Background/Objectives: The rapid evolution of severe acute respiratory syndrome coronavirus 2 (SARS-CoV-2) has led to the emergence of variants with enhanced transmissibility and immune evasion, challenging existing vaccines. This study aimed to evaluate the immunogenicity and protective efficacy of inactivated bivalent vaccine formulations incorporating the ancestral SARS-CoV-2 strain (ERAGEM) with either Delta or Omicron (BA.5) variants. Methods: Bivalent vaccine formulations were prepared using beta-propiolactone-inactivated SARS-CoV-2 antigens and administered to K18-hACE2 transgenic mice. Following prime and booster immunizations, neutralizing antibody titers and viral loads were assessed through ELISA, microneutralization assays, and quantitative PCR. Mice were challenged with the respective variants, and the survival rates, temperature, and body weight changes were monitored for 21 days. Results: Both vaccine formulations elicited significant increases in neutralizing antibody titers post-booster immunization. The ERAGEM + Delta group demonstrated geometric mean titers (GMTs) of 6938.1 and 4935.0 for the ancestral and Delta variants, respectively, while the ERAGEM + Omicron (BA.5) group achieved GMTs of 16,280.7 and 24,215.9 for the ancestral and Omicron (BA.5) variants. Complete survival (100%) was observed in all the vaccinated groups post-challenge, with no detectable viral titers in the lungs and substantial reductions in the nasal turbinate viral loads compared to the unvaccinated controls. Conclusions: The bivalent inactivated vaccines demonstrated strong immunogenicity and complete protection against severe disease in preclinical models. These findings indicate the potential of bivalent vaccine strategies in addressing antigenic diversity and preparing for future pandemics caused by rapidly evolving pathogens.

## 1. Introduction

Coronaviruses represent a diverse family of enveloped, positive-sense, single-stranded RNA viruses, distinguished by their extensive genomes of approximately 30 kilobases, making them among the largest known RNA viruses [1]. These viruses are characterized structurally by their spike (S) proteins, which exhibit a crown-like appearance under electron microscopy and mediate viral entry into host cells via the angiotensin-converting enzyme 2 (ACE2) receptor [2,3]. Other structural proteins, including membrane (M), envelope (E), and nucleocapsid (N) proteins, are crucial for viral assembly, release, and RNA binding, respectively. [1]. Coronaviruses are divided into four genera—*Alphacoronavirus*, *Betacoronavirus*, *Gammacoronavirus*, and *Deltacoronavirus*—each associated with distinct host ranges and pathogenic profiles [4,5]. Among these, Betacoronaviruses include highly pathogenic species such as severe acute respiratory syndrome coronavirus 1 (SARS-CoV-1), Middle East respiratory syndrome coronavirus (MERS-CoV), and severe acute respiratory syndrome coronavirus 2 (SARS-CoV-2), the causative agent of the COVID-19 pandemic [6,7].

SARS-CoV-2, first identified in Wuhan, China, in December 2019, rapidly became a global threat due to its efficient human-to-human transmission [8]. The rapid evolution of SARS-CoV-2 has posed a significant challenge to global public health, leading to the emergence of multiple variants of concern (VOCs) [9,10]. The urgency of the COVID-19 pandemic has accelerated vaccine development, with several platforms rapidly advancing through clinical trials. Nucleic acid vaccines (DNA and RNA) and viral vector vaccines have emerged as leading candidates due to their adaptability and ability to induce strong immune responses. RNA vaccines developed by Moderna and Pfizer-BioNTech, which encode the spike protein, have shown high efficacy in clinical trials [11,12]. Similarly, viral vector vaccines, such as AstraZeneca’s ChAdOx1 and Johnson & Johnson’s Ad26, have demonstrated promising results in eliciting both humoral and cellular immunity [13,14]. Subunit vaccines, such as those developed by Novavax, utilize adjuvanted spike proteins to enhance immunogenicity, yielding strong antibody responses in clinical studies [15]. Additionally, inactivated vaccines, such as those from Sinopharm, Sinovac, and TURKOVAC, have also shown promise; these vaccines, which use chemically inactivated SARS-CoV-2, have demonstrated safety and the ability to induce an immune response [16,17,18,19].

The rapid evolution of SARS-CoV-2 variants and the reduced efficacy of existing vaccines underscore the urgent need for next-generation vaccine platforms that are capable of addressing these challenges. This evolution is driven by a combination of genetic characteristics, host interactions, environmental pressures, and specific mutational events [20,21]. Among these, mutations in the viral spike protein, a critical structure that binds to the ACE2 receptor in human tissues, have been particularly impactful. These mutations enhance viral infectivity and facilitate immune evasion, significantly increasing transmissibility and enabling the virus to bypass neutralizing antibodies [20,22]. The high human-to-human transmission efficiency, coupled with super-spreading events, has further amplified the virus’s global dissemination and mutation accumulation [23,24]. SARS-CoV-2 variants have demonstrated diverse genetic adaptations, with key VOCs exhibiting unique mutational profiles that influence their transmissibility and immune escape capabilities. The Alpha variant (B.1.1.7), first identified in the UK in September 2020, became dominant due to its increased transmissibility, driven by mutations such as N501Y and D614G, which enhance infectivity and stabilize the spike protein [25,26]. The Beta variant (B.1.351), detected in South Africa in May 2020, raised significant concerns about immune evasion through mutations such as K417N and E484K, which impair antibody binding and neutralization [27]. Similarly, the Gamma variant (P.1), identified in Brazil in November 2020, shares immune-evasive mutations with Beta, further enhancing its transmissibility [28,29]. The Delta variant (B.1.617.2), first reported in India in October 2020, exhibited mutations such as L452R and P681R, contributing to its rapid global spread and immune evasion [30,31]. Most recently, the Omicron variant (B.1.1.529), identified in South Africa in November 2021, demonstrated extensive spike protein mutations, significantly enhancing transmissibility and immune escape. Omicron’s subvariants have introduced additional mutations that increase ACE2 binding affinity and immune evasion [32,33,34,35].

Multivalent vaccine strategies, which target multiple viral strains, hold great potential for providing broad-spectrum protection against the diverse and continuously evolving variants of SARS-CoV-2. The aim of this study is to develop and evaluate a bivalent inactivated vaccine that targets distinct SARS-CoV-2 variants to achieve enhanced and broad-spectrum immunological protection. This study focuses on formulating bivalent vaccines that incorporate antigens from the ancestral SARS-CoV-2 strain in combination with either the Delta or Omicron (BA.5) variants. The objective is to broaden the immune response and enhance protection against multiple SARS-CoV-2 variants. By addressing the limitations of existing monovalent vaccines, this study aims to demonstrate the critical role of bivalent vaccine strategies in eliciting wide-ranging immunity. Such an approach is essential for controlling the evolving epidemiology of COVID-19 and mitigating the impact of emerging variants, ensuring comprehensive protection against the dynamic challenges posed by SARS-CoV-2.

## 2. Materials and Methods

### 2.1. Cell and Viruses

Vero An16 cells (African green monkey kidney), acquired from the American Type Culture Collection (ATCC) (CRL-1586), were maintained in Dulbecco’s Modified Eagle’s Medium (DMEM) enriched with 10% heat-inactivated fetal bovine serum (FBS), 100 mM of L-glutamine, 100 U/mL of penicillin, and 100 µg/mL of streptomycin. The ancestral SARS-CoV-2 strain (hCoV-19/Turkey/ERAGEM-001/2020) was originally isolated and characterized by our group in Turkey during the early phase of the pandemic [36]. The SARS-CoV-2 Delta variant was isolated from nasal swab samples collected from COVID-19-positive patients at Kayseri City Hospital. The SARS-CoV-2 Omicron (BA.5) variant was obtained from the Turkish Ministry of Health, General Directorate of Public Health. Comprehensive sequencing and subsequent translation confirmed the complete genomic sequences of the SARS-CoV-2 variants. The ancestral SARS-CoV-2 strain (hCoV-19/Turkey/ERAGEM-001/2020) was determined to have a complete genome size of 29,832 bp and has been deposited in GenBank under accession number MT327745.1. The Delta variant exhibited a genome size of 29,849 bp (GenBank accession OM945721). The Omicron (BA.5) variant was found to have a complete genome size of 29,831 bp, with the sequence available under GISIAD accession number EPI_ISL_14394492.1. The viral titers of the SARS-CoV-2 strains were determined using the tissue culture infective dose 50% (TCID_50_) method [36].

### 2.2. Facility and Ethics Statement

All procedures involving SARS-CoV-2 were carried out in a certified biosafety level 3 (BSL-3) laboratory at the Erciyes University Vaccine Research and Development Center (ERAGEM). The laboratory operates under strict national and international biosafety regulations, ensuring a secure environment for working with high-containment pathogens. The animal studies were approved by the Erciyes University Animal Experiments Local Ethics Committee (Approval No. 22/144) and followed established ethical guidelines to ensure humane treatment. All experimental protocols were designed to align with the internationally accepted standards, including measures to minimize discomfort and distress in laboratory animals.

### 2.3. Animals

Six-week-old female K18-hACE2 transgenic mice (B6.Cg-Tg(K18-hACE2)2Prlmn/J) were purchased from Jackson Laboratories (Bar Harbor, ME, USA). The mice were maintained in a controlled environment at 20–22 °C with a relative humidity of 50 ± 10% on a 12 h light/dark cycle, with free access to rodent chow and tap water. All animal care and experiments adhered to the guidelines for animal protection and laboratory animal use as outlined in Regulation 5199, which governs animal welfare and research practices in Türkiye.

### 2.4. Vaccine Antigens and Vaccinations of Mice

**Figure 1:**
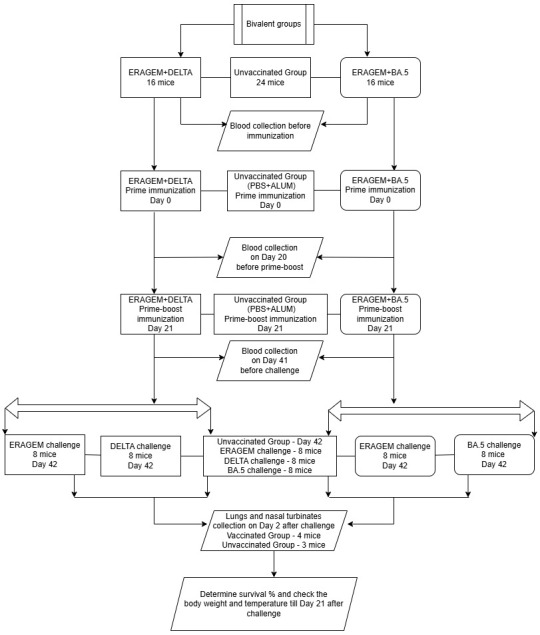
Experimental design for immunization and challenge of K18-hACE2 transgenic mice in bivalent vaccine groups. The figure describes the immunization and challenge protocol for mice vaccinated with bivalent formulations of severe acute respiratory syndrome coronavirus 2 (SARS-CoV-2) strains (ERAGEM + Delta and ERAGEM + Omicron (BA.5)). Blood sampling was performed at specified intervals, and lung and nasal turbinate tissues were collected post-challenge to assess immune responses and viral replication.

Vero An16 cells were cultured in DMEM supplemented with 10% heat-inactivated fetal bovine serum (FBS), 100 mM of L-glutamine, 100 U/mL of penicillin, and 100 µg/mL of streptomycin and infected with the SARS-CoV-2 variants ERAGEM, Delta, and Omicron (BA.5) at a multiplicity of infection of 0.001. Following infection, the Vero An16 cells were cultured for 72 h before harvesting, and the supernatant was collected and centrifuged to remove cellular debris. The viral bulk culture was precipitated using 0.5 M NaCl, followed by the addition of 10% (*w*/*v*) PEG 8000, and the mixture was stirred overnight at 4 °C. Afterward, the solution was centrifuged at 12,000 rpm for 30 min at 4 °C. The supernatant was discarded, and the viral pellet was resuspended in TNE buffer (50 mM Tris-HCl, 150 mM NaCl, and 5 mM EDTA, pH 7.2). To further purify the virus, a sucrose gradient was utilized to ensure high-quality preparations. The viral solution was applied to a 20–60% sucrose gradient and centrifuged using a Beckman SW28 rotor at 25,000 rpm at 4 °C. Fractions of 500 µL were collected from the bottom of the centrifuge tube using a fraction collector. The virus fractions were then diluted four-fold with PBS and subjected to ultracentrifugation at 24,000 rpm at 4 °C. The resulting virus pellets were resuspended in 1 mL of PBS. The antigen content of the purified product was quantified using the Lowry protein assay. The antigen doses for the vaccine formulations were determined to consist of 1.5 µg of ERAGEM combined with 1.5 µg of Delta and 1.5 µg of ERAGEM combined with 1.5 µg of Omicron (BA.5). The inactivated and purified vaccine antigens were formulated to contain a total of 3 µg of antigen per dose, along with 250 µg of aluminum hydroxide (Alhydrogel, 250 µg per dose) (InvivoGen, San Diego, CA, USA) as an adjuvant in a 250 µL volume. The total antigen content was quantified prior to formulation to ensure consistency and accuracy in the dose preparation as previously described [37]. A total of 56 mice were used in the study, divided into two vaccine groups and one negative control group: 16 mice were allocated to the ERAGEM + Delta group, 16 mice to the ERAGEM + Omicron (BA.5) group, and 24 mice to the negative control group. In the vaccinated groups, each mouse received an intraperitoneal injection containing 1.5 µg of antigen from each variant in the respective group. Baseline antibody levels were measured from the blood samples collected prior to immunization. Both groups were administered a prime immunization on Day 0. A second blood collection was performed on Day 20 to assess the immune responses before administering the booster immunization on Day 21, as shown in Figure 1. Subsequently, blood samples were collected again on Day 41 to evaluate the antibody responses prior to the challenge phase. On Day 42, mice in the ERAGEM + Delta group were further subdivided, with 8 mice challenged with the ERAGEM variant and 8 challenged with the Delta variant, using a viral titer of 5 × 10^4^ TCID_50_ as described previously [37]. Similarly, in the ERAGEM + Omicron (BA.5) group, 8 mice were challenged with the ERAGEM variant, and the remaining 8 were challenged with the Omicron (BA.5) variant, also using a viral titer of 5 × 10^4^ TCID_50_ (Figure 1). Two days post-challenge, lung and nasal turbinate tissues were collected to assess the viral titers and loads as described previously [37]. Following the challenge, the mice were monitored daily for survival, body weight, and temperature changes over a 21-day period. Antibody levels in the blood samples collected at baseline, after the prime immunization, and after the prime-boost immunization were quantified using ELISA and MN_50_ assays. The viral titers and loads in lung and nasal turbinate tissues were evaluated using focus-forming unit (FFU) assays and quantitative PCR (qPCR) (Figure 1). Data on the survival rates, body weight changes, and temperature variations were subjected to statistical analysis to determine vaccine efficacy.

### 2.5. Virus Titer and Fluorescent Focus Assay

To determine the viral titers, lung and nasal turbinate tissues were homogenized in 1 mL of DMEM that contained antibiotics (100 U/mL of penicillin and 100 µg/mL of streptomycin). The homogenates were centrifuged at 18,000× *g* for 10 min, and the supernatants were carefully collected. The resulting supernatants were used to inoculate confluent Vero cell monolayers, which were incubated at 37 °C for 1 h to facilitate viral adsorption. After removing the inoculum, the monolayers were washed with phosphate-buffered saline (PBS) and overlaid with a medium containing 1% carboxymethyl cellulose (CMC) to support plaque formation. The plates were incubated at 37 °C with 5% CO_2_ for 72 h. Subsequent to the incubation, the cells were fixed using 10% neutral-buffered formaldehyde and permeabilized with 0.1% Triton X-100 in PBS. Blocking was conducted with 5% skim milk prepared in PBS. The plates were then incubated with a human anti-SARS-CoV-2 nucleocapsid protein antibody at a 1:2500 dilution. After washing, a secondary antibody conjugated with fluorescein isothiocyanate (FITC) was applied at a 1:1000 dilution. Excess antibodies were removed with multiple washes using tris-buffered saline with 0.1% Tween-20 and distilled water. Fluorescent foci were visualized and quantified using fluorescence microscopy, and the viral titers were expressed in fluorescent focus units (FFUs) per milliliter [37].

### 2.6. Quantitative Polymerase Chain Reaction (qPCR)

To prepare the samples for analysis, lung and nasal turbinate tissues were homogenized in 1 mL of DMEM supplemented with antibiotics (penicillin at 100 U/mL and streptomycin at 100 µg/mL). The homogenates were centrifuged at 18,000× *g* for 10 min at 4 °C to separate cellular debris, and the supernatants were collected. RNA extraction was carried out using the QIAamp Viral RNA Mini Kit (Qiagen, Germany) according to the manufacturer’s instructions, starting with 140 µL of each processed sample. Quantification of SARS-CoV-2 nucleocapsid (N) RNA was performed using the Diagnovital SARS-CoV-2 Real-Time PCR Kit v2.0 (RTA Laboratories, Istanbul, Turkey) on a Rotor-Gene Q thermocycler (Qiagen, Germany) according to the manufacturer’s instructions. A standard curve was generated by serially diluting the SARS-CoV-2 RNA reference standards with known copy numbers, enabling accurate viral load calculations [37]. The results are expressed as RNA copy numbers per milliliter of the original homogenate, allowing for consistent and precise comparisons across all the experimental groups.

### 2.7. Enzyme-Linked Immunosorbent Assay (ELISA)

SARS-CoV-2 antigens from ancestral and variant strains were prepared using established purification techniques, including sucrose gradient ultracentrifugation. The ELISA plates (Maxisorp, Nunc, UK) were coated overnight at 4 °C with 1 µg/well of purified antigen in carbonate buffer (pH 9.4). After washing with PBS containing 0.05% Tween-20, the plates were blocked with 5% skim milk for 1 h at room temperature to prevent nonspecific binding. Serum samples were diluted at 1:100 as a starting point, followed by serial two-fold dilutions. The diluted sera were added to the antigen-coated wells and incubated for 1 h at 37 °C. After washing, horseradish peroxidase (HRP)-conjugated goat anti-mouse IgG (diluted 1:2000; Southern Biotech, USA) was applied and incubated for an additional hour at 37 °C. Substrate solution (TMB; Kementec, Denmark) was added to each well and incubated in the dark for 15 min. The enzymatic reaction was terminated with 2 M sulfuric acid, and the absorbance was measured at 450 nm using a spectrophotometer (Biotek ELx80, Thermo Fisher Scientific, UK). The optical density (OD) cutoff value was defined as three times the mean OD of the negative control wells. The endpoint titers were calculated based on the highest serum dilution exceeding the cutoff. The results are expressed as geometric mean titers (GMTs) ± standard error (SE), providing a robust assessment of the humoral immune responses.

### 2.8. Microneutralization Test (MNT)

The microneutralization test (MNT) was conducted to measure SARS-CoV-2-specific neutralizing antibodies. The serum samples were heat-inactivated at 56 °C for 1 h before testing. The serum samples were initially diluted at 1:2 and subjected to serial two-fold dilutions. Each diluted serum sample (50 µL) was mixed with an equal volume (50 µL) of SARS-CoV-2 containing 100 TCID_50_, resulting in a total reaction volume of 100 µL. The mixtures were incubated for 1 h at 37 °C to allow neutralization. The mixtures were then added to confluent Vero E6 cell monolayers in 96-well plates. After removing the inoculum, 150 µL of DMEM supplemented with 2% FBS was added to each well. Each serum dilution was tested in triplicate, with six wells as the virus controls and three blank wells containing only growth medium. Cytopathic effects were observed daily, with a final evaluation at 3–4 days post-infection (dpi). The 50% neutralization titer (NT_50_) was defined as the highest serum dilution that prevented the cytopathic effects in 50% of the wells. A titer of ≥1/8 was considered indicative of seropositivity.

### 2.9. Statistical Analysis

Statistical analyses were performed using GraphPad Prism (Version 10, GraphPad Software, San Diego, CA, USA) and Microsoft Excel 2019 (Microsoft). Kaplan–Meier survival curves were generated to depict the survival outcomes, and the log-rank test was used to compare the survival rates following the viral challenge. Body weight and temperature data were analyzed using Excel and visualized in GraphPad Prism. Differences between groups in ELISA were assessed by a Student’s *t*-test, and microneutralization assays were assessed using the Mann–Whitney *U* test. For FFUs and RNA copy numbers, an unpaired Student’s *t*-test was used to evaluate the differences between groups. Statistical significance was indicated as follows: * *p* < 0.05, ** *p* < 0.005, *** *p* < 0.0005, and **** *p* < 0.0001.

## 3. Results

### 3.1. Bivalent Vaccines Protect K18-hACE2 Transgenic Mice Against the Lethal SARS-CoV-2 Variants Challenge

**Figure 2:**
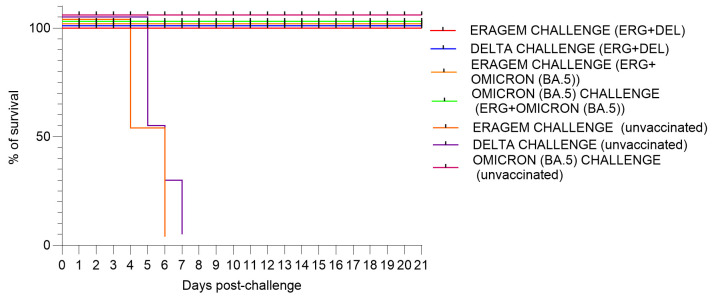
Survival analysis of mice vaccinated with bivalent severe acute respiratory syndrome coronavirus 2 (SARS-CoV-2) vaccines (ERAGEM + Delta and ERAGEM + Omicron (BA.5)) followed by challenge with the respective SARS-CoV-2 variants. The figure shows the survival percentages of mice over a 21-day period after being challenged with SARS-CoV-2 variants. Mice vaccinated with the ERAGEM + Delta bivalent group (ERG + DEL) were challenged with the ERAGEM variant (red curve) and the Delta variant (blue curve), while those vaccinated with the ERAGEM + Omicron (BA.5) bivalent group (ERG + Omicron (BA.5)) were challenged with the ERAGEM variant (orange curve) and the Omicron (BA.5) variant (green curve). The negative control groups for each variant are represented as follows: ERAGEM (brown), Delta (purple), and Omicron (BA.5) (pink). These survival curves provide an evaluation of the protective efficacy of the bivalent vaccines against SARS-CoV-2 variants based on survival outcomes in each experimental group.

This study evaluated the efficacy of bivalent vaccines in mice immunized with ERAGEM + Delta or ERAGEM + Omicron (BA.5) and subsequently challenged with their respective variants. Mice vaccinated with the ERAGEM + Delta combination demonstrated 100% survival when challenged with either the ERAGEM or Delta variants over the 21-day observation period. Similarly, mice immunized with the ERAGEM + Omicron (BA.5) combination exhibited 100% survival following the challenges with both the ERAGEM and Omicron (BA.5) variants (Figure 2). In contrast, the unvaccinated control group showed a significantly different outcome. All mice in this group succumbed to infection within 6 to 7 days following challenges with the ERAGEM and Delta variants, emphasizing the high vulnerability of unvaccinated mice to infections caused by these variants (Figure 2). Notably, the absence of mortality in the control group challenged with the Omicron (BA.5) variant indicates a lower pathogenicity of Omicron (BA.5) compared to the ERAGEM and Delta variants (Figure 2).

These results highlight the significant efficacy of the bivalent vaccines in preventing mortality. The protective effects observed in these groups emphasize the potential of these bivalent vaccines to confer broad-spectrum immunity against multiple SARS-CoV-2 variants.

**Figure 3:**
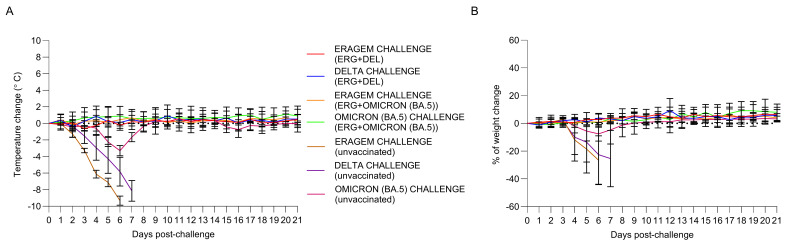
Evaluation of temperature and weight changes in mice vaccinated with bivalent vaccines (ERAGEM + Delta and ERAGEM + Omicron (BA.5)) prior to challenge with the respective SARS-CoV-2 variants. (**A**) The longitudinal analysis of temperature variations following viral challenge shows the mean temperature changes (± SEM) recorded in mice challenged with ERAGEM (red curve) and Delta (blue curve) in the ERAGEM + Delta bivalent vaccine group (ERG + DEL), as well as ERAGEM (orange curve) and Omicron (BA.5) (green curve) in the ERAGEM + Omicron (BA.5) bivalent vaccine group (ERG + Omicron (BA.5)). Negative control groups are presented for ERAGEM (purple curve), Delta (brown curve), and Omicron (BA.5) (pink curve) for comparison. (**B**) The longitudinal analysis of weight variations post-viral challenge depicts the mean percentage weight changes (± SEM) in the same experimental groups. Negative control groups for ERAGEM (purple curve), Delta (brown curve), and Omicron (BA.5) (pink curve) are included. Data were collected daily for 21 days following the challenge to assess the physiological impacts of the viral infection and vaccine efficacy.

Temperature variations in the mice following the viral challenge are presented in Figure 3A. Mice vaccinated with the ERAGEM + Delta and ERAGEM + Omicron (BA.5) bivalent vaccines maintained stable temperatures throughout the 21-day post-challenge period, showing no significant deviations. In contrast, the negative control groups exhibited a marked decline in temperature starting on Day 2 post-challenge, with the most substantial drop occurring around Day 6. This temperature decline in the control groups reflects the severity of the infection and impending mortality. Body weight changes in the mice over the same period are shown in Figure 3B. Vaccinated mice in the ERAGEM + Delta and ERAGEM + Omicron (BA.5) groups showed relatively stable body weights, with only minimal fluctuations during the 21-day post-challenge period. In comparison, the negative control groups experienced significant weight loss beginning on Day 2 post-challenge, with a pronounced decrease observed by Day 6. This weight loss further confirms the severe impact of the viral infection in unvaccinated mice. The stability of both temperature and body weight in the vaccinated groups (Figure 3A,B) demonstrates the protective efficacy of the bivalent vaccines. Vaccinated mice were able to maintain normal physiological parameters, indicative of effective protection against the viral challenge. In contrast, the significant declines in temperature and body weight in the negative control groups underscore the severity of the infection in the absence of vaccination.

### 3.2. Bivalent Vaccines Induce Humoral Immune Responses in K18-hACE2 Transgenic Mice

**Figure 4:**
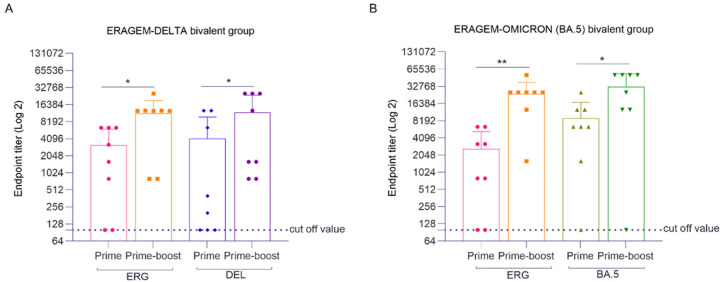
Endpoint antibody titers in mice vaccinated with bivalent ERAGEM + Delta and ERAGEM + Omicron (BA.5) vaccines followed by booster immunization. The figure shows the endpoint antibody titers for the ERAGEM + Delta (**A**) and ERAGEM + Omicron (BA.5) (**B**) bivalent vaccine groups at two time points: after the initial immunization (prime) and following the booster dose (prime-boost). In (**A**), the pink bar represents ERAGEM for the prime immunization, the orange bar represents ERAGEM for the prime-boost immunization, the blue bar represents Delta for the prime immunization, and the purple bar represents Delta for the prime-boost immunization. In (**B**), the pink bar represents ERAGEM for the prime immunization and the orange bar represents ERAGEM for the prime-boost immunization, while the green bar represents Omicron (BA.5) for the prime immunization and the dark green bar represents Omicron (BA.5) for the prime-boost immunization. The ELISA plates were coated with antigens corresponding to each variant: ERG for ERAGEM antigen coating, DEL for Delta antigen coating, and BA.5 for Omicron (BA.5) antigen coating. The dashed lines indicate the cutoff values for antibody detection, and the error bars represent the standard errors of the means (SEMs). Statistical significance is indicated as * *p* < 0.05 and ** *p* < 0.005. These findings highlight the enhanced antibody response against both the ancestral and variant strains following booster immunization in both bivalent vaccine groups.

In the ERAGEM + Delta bivalent vaccine group, endpoint titers for the ERAGEM variant significantly increased between the first immunization and the prime-boost one (* *p* < 0.05), reflecting a substantial enhancement in the antibody response following the booster dose. The GMT increased from 1345.4 after the first immunization to 6938.1 after the booster, demonstrating a strong improvement in the immune response (Figure 4A). Similarly, for the Delta variant, the endpoint titers significantly increased between the first immunization and the booster (* *p* < 0.05). The GMT rose from 733.6 following the first immunization to 4935.0 after the booster (Figure 4A). Collectively, these findings indicate that the ERAGEM + Delta bivalent vaccine effectively enhanced the immune response against both the ERAGEM and Delta variants after the booster dose. In the ERAGEM + Omicron (BA.5) bivalent vaccine group, the endpoint titers for the ERAGEM variant also significantly increased following the booster immunization (** *p* < 0.005), with the GMTs rising from 841.5 to 16,280.7, highlighting a marked enhancement in the antibody response (Figure 4B). Likewise, for the Omicron (BA.5) variant, the endpoint titers significantly increased after the booster immunization (* *p* < 0.05), with the GMTs increasing from 11,800.4 to 24,215.9. These results suggest that the booster immunization elicited a strong immune response against the Omicron (BA.5) variant (Figure 4B). Overall, both the ERAGEM + Delta and ERAGEM + Omicron (BA.5) bivalent vaccine groups demonstrated significant increases in the endpoint titers following the booster immunization, indicating a substantial improvement in the antibody response (Figure 4A,B).

**Figure 5:**
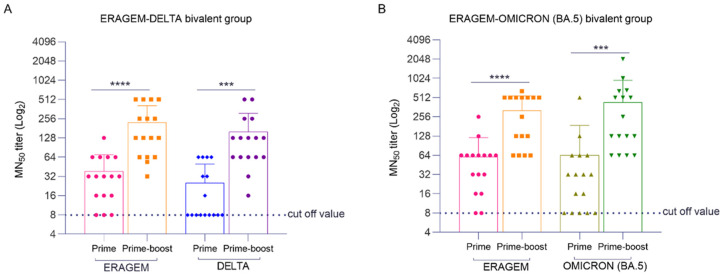
Neutralizing antibody response in mice vaccinated with bivalent vaccines (ERAGEM + Delta and ERAGEM + Omicron (BA.5)) followed by prime and prime-boost immunizations. Neutralizing antibody responses were evaluated using sera from bivalent vaccine groups against ERAGEM, Delta, and Omicron (BA.5) variants. (**A**) For the ERAGEM + Delta bivalent group, neutralization was assessed against ERAGEM and Delta variants. (**B**) For the ERAGEM + Omicron (BA.5) bivalent group, sera were tested for neutralization of ERAGEM and Omicron (BA.5) variants. In both panels, the pink bars represent neutralization titers against ERAGEM after the prime immunization, and the orange bars represent neutralization titers against ERAGEM after the prime-boost immunization. The blue bars represent neutralization titers against the Delta variant after the prime immunization, and the purple bars represent titers against the Delta variant after the prime-boost immunization (**A**). The green bars represent neutralization titers against Omicron (BA.5) after the prime immunization, while the dark green bars represent titers against Omicron (BA.5) after the prime-boost immunization (**B**). The dashed lines indicate the cutoff values for neutralization titers, and the error bars represent the standard error of the mean (SEM). Statistical analysis was performed using an unpaired *t*-test to evaluate differences between prime and prime-boost responses, with *** *p* < 0.001 and **** *p* < 0.0001 considered significant.

To evaluate the neutralizing antibody titers in the bivalent vaccine groups, microneutralization (MN_50_) assays were performed. In the ERAGEM + Delta bivalent vaccine group (Figure 5A), a significant increase in MN_50_ titers was observed following the booster immunization compared to the first immunization. Specifically, the MN_50_ titers against ERAGEM increased from a GMT of 28.10 after the first dose to 157.1 following the booster dose (**** *p* < 0.0001). Similarly, the MN_50_ titers against Delta increased substantially, with the GMTs rising from 16.7 to 107.6 (*** *p* < 0.001), indicating a significant enhancement in the immune response. In the ERAGEM + Omicron (BA.5) bivalent vaccine group (Figure 5B), the booster immunization also resulted in significantly higher MN_50_ titers compared to the first dose. The MN_50_ titers against ERAGEM increased, with the GMTs rising from 41.50 to 228.09 (**** *p* < 0.0001) after the booster. Additionally, the MN_50_ titers against Omicron (BA.5) significantly increased, with the GMTs rising from 28.10 to 242.15 (*** *p* < 0.001), demonstrating an improved and effective immune response. These findings highlight that the booster immunization strategy significantly enhances the neutralizing antibody response in both the ERAGEM + Delta and ERAGEM + Omicron (BA.5) bivalent vaccine groups. The observed increases in the MN_50_ titers following the booster regimen suggest that these bivalent vaccines effectively induce potent neutralizing activity against the ERAGEM, Delta, and Omicron (BA.5) variants of SARS-CoV-2.

### 3.3. Bivalent Vaccines Provide Complete Inhibition of Viral Replication in the Lungs and a Significant Reduction in Nasal Turbinates

**Figure 6:**
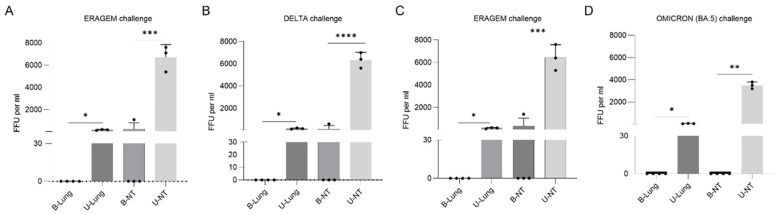
Viral titers in lung and nasal turbinate samples of mice immunized with bivalent vaccines following SARS-CoV-2 challenges. Viral titers in lung and nasal turbinate samples were evaluated two days post-challenge with SARS-CoV-2 variants (ERAGEM, Delta, and Omicron (BA.5)) in mice immunized with bivalent vaccines (ERAGEM + Delta; ERAGEM + Omicron (BA.5)). Viral loads, measured as fluorescent focus units per milliliter (FFU/mL), were determined using an immunofluorescence assay. (**A**,**B**) depict the results from the ERAGEM + Delta bivalent vaccine group following challenges with ERAGEM (**A**) and Delta (**B**). (**C**,**D**) show results from the ERAGEM + Omicron (BA.5) bivalent vaccine group challenged with ERAGEM (**C**) and Omicron (BA.5) (**D**). Experimental groups include lung samples from immunized (B-Lung) and unvaccinated control mice (U-Lung), as well as nasal turbinate samples from immunized (B-NT) and unvaccinated control mice (U-NT). Scatter plots and bar graphs illustrate viral titers across groups. Statistical analyses were performed using unpaired t-tests, with significance thresholds at * *p* < 0.05, ** *p* < 0.005, *** *p* < 0.0005 and **** *p* < 0.0001. GraphPad Prism version 10 software was used for data analysis and visualization.

To evaluate the efficacy of the bivalent vaccine groups, the viral titers were measured in the lungs and nasal turbinates of four mice from each vaccine group and three mice from the unvaccinated control group on the second day post-challenge. In the ERAGEM + Delta bivalent vaccine group, viral replication was detected in the nasal turbinates of only one of four mice following challenges with the ERAGEM and Delta variants, whereas no viral replication was observed in the other three mice. For the ERAGEM challenge in this group, no viral titers were detected in the lungs (Bivalent-L), while the unvaccinated control group exhibited 172.4 FFU/mL in the lungs (Bivalent NC-L). In the nasal turbinates, the viral titers were 400.2 FFU/mL in the bivalent group (Bivalent-NT) compared to 6629.6 FFU/mL in the unvaccinated control group (Bivalent NC-NT) (Figure 6A). For the Delta challenge, no viral titers were detected in the lungs of the bivalent group (Bivalent-L), while the unvaccinated control group displayed 166.4 FFU/mL (Bivalent NC-L). In the nasal turbinates, the viral titers were 485.2 FFU/mL in the bivalent group (Bivalent-NT) and 6306.9 FFU/mL in the unvaccinated control group (Bivalent NC-NT) (Figure 6B).

In the ERAGEM + Omicron (BA.5) bivalent vaccine group, following an ERAGEM challenge (Figure 6C), no viral titers were detected in the lungs of the bivalent group (Bivalent-L), while the unvaccinated control group exhibited 153.94 FFU/mL (Bivalent NC-L). In the nasal turbinates, the titers were 950 FFU/mL in the bivalent group (Bivalent-NT) and 6364.39 FFU/mL in the unvaccinated control group (Bivalent NC-NT). For the Omicron (BA.5) challenge, no viral replication was detected in any mice immunized with the ERAGEM + Omicron (BA.5) bivalent vaccine. The viral titers for the Omicron (BA.5) challenge group were as follows: no viral titers detected in the lungs (Bivalent-L), 40.1 FFU/mL in the lungs of the unvaccinated control group (Bivalent NC-L), no viral titers detected in the nasal turbinates (Bivalent-NT), and 3497.4 FFU/mL in the nasal turbinates of the unvaccinated control group (Bivalent NC-NT) (Figure 6D).

These results demonstrate that the ERAGEM + Delta bivalent vaccine provides complete protection in the lungs following ERAGEM and Delta challenges. However, limited viral replication in the nasal turbinates of one mouse suggests a degree of residual susceptibility in nasal tissues. In contrast, the ERAGEM + Omicron (BA.5) bivalent vaccine provided complete protection in both the lungs and nasal turbinates against the Omicron (BA.5) challenge, with no viral replication detected. The absence of viral titers in the Omicron (BA.5) challenge group, compared to the presence of viral titers in the nasal turbinate samples following ERAGEM and Delta challenges, may reflect lower virulence of the Omicron (BA.5) variant relative to the ERAGEM and Delta variants.

**Figure 7:**
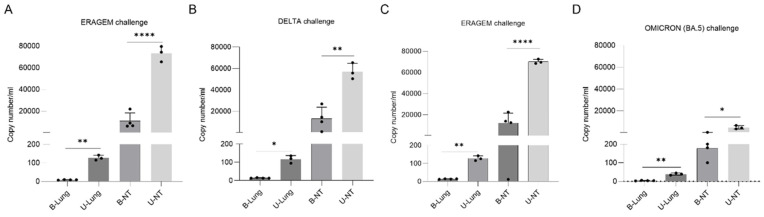
Viral copy numbers in lung and nasal turbinate samples of mice immunized with bivalent vaccines followed by SARS-CoV-2 challenges. Viral copy numbers in lung (L) and nasal turbinate (NT) samples were measured in mice immunized with bivalent vaccines (ERAGEM + Delta; ERAGEM + Omicron (BA.5)) two days post-challenge with SARS-CoV-2 variants (ERAGEM, Delta, and Omicron (BA.5)). (**A**–**D**) show viral copy numbers for ERAGEM, Delta, ERAGEM, and Omicron (BA.5) challenges, respectively. The following abbreviations are used: B-Lung for lung samples from immunized mice, U-Lung for lung samples from unvaccinated negative control mice, B-NT for nasal turbinate samples from immunized mice, and U-NT for nasal turbinate samples from unvaccinated negative control mice. Data are presented as scatter plots and bar graphs to indicate viral loads across experimental groups. An unpaired *t*-test was used to determine statistically significant differences between groups, with statistical significance indicated as * *p* < 0.05, ** *p* < 0.005, and **** *p* < 0.0001. All analyses and graphical representations were performed using GraphPad Prism version 10 software.

After determining the viral titers using FFUs, the viral RNA levels were measured in lung and nasal turbinate samples collected two days after the SARS-CoV-2 challenges. Real-time PCR was used to quantify the viral RNA copy numbers in the mice immunized with the ERAGEM + Delta or ERAGEM + Omicron (BA.5) vaccines, as well as in the unvaccinated controls. In the ERAGEM + Delta bivalent group, the viral RNA levels following the ERAGEM challenge (Figure 7A) were 14.6 copies/mL in the lungs of the vaccinated mice compared to 126.8 copies/mL in the unvaccinated controls. In the nasal turbinates, the vaccinated mice had 9361.9 copies/mL, significantly lower than the 72,858.6 copies/mL in the unvaccinated controls (** *p* < 0.005; **** *p* < 0.0001). For the Delta challenge (Figure 7B), the vaccinated mice had 13.69 copies/mL in the lungs versus 115.4 copies/mL in the unvaccinated controls. In the nasal turbinates, the vaccinated mice had 7707.4 copies/mL compared to 56,718.5 copies/mL in the unvaccinated controls (* *p* < 0.05; ** *p* < 0.005; **** *p* < 0.0001).

In the ERAGEM + Omicron (BA.5) bivalent group, the viral RNA levels after the ERAGEM challenge (Figure 7C) were undetectable in the lungs of the vaccinated mice, while the unvaccinated controls had 153.94 copies/mL. In the nasal turbinates, the vaccinated mice had 950 copies/mL compared to 6364.39 copies/mL in the unvaccinated controls (** *p* < 0.005; **** *p* < 0.0001). Following the Omicron (BA.5) challenge (Figure 7D), the vaccinated mice exhibited 18.2 copies/mL in the lungs versus 39 copies/mL in the unvaccinated controls. In the nasal turbinates, the vaccinated mice had 183.7 copies/mL compared to 4610.9 copies/mL in the unvaccinated controls (* *p* < 0.05; ** *p* < 0.005).

These results demonstrate that both bivalent vaccine groups effectively reduced viral replication in the lungs and nasal turbinates compared to the unvaccinated controls. The ERAGEM + Omicron (BA.5) vaccine provided substantial protection, with minimal viral RNA detected in both tissue types, while the Omicron (BA.5) challenge exhibited lower overall viral RNA levels compared to other variants.

## 4. Discussion

The COVID-19 pandemic has significantly affected global public health and has led to widespread social, cultural, and economic disruptions [38]. Since its initial identification in December 2019 in Wuhan, China, the virus has spread worldwide, resulting in millions of infections and widespread disruptions. By 2024, over 775 million confirmed cases and more than 7 million deaths had been reported globally, highlighting the extensive public health and societal burden of the pandemic [39]. In response to the devastating impacts of the pandemic, scientists worldwide rapidly initiated SARS-CoV-2 vaccine development. During the COVID-19 pandemic, multiple vaccine platforms were established to address the transmission of SARS-CoV-2. These included inactivated, mRNA, viral vector, and protein subunit vaccines, each contributing to the global vaccination effort [40,41,42,43]. Among these platforms, inactivated vaccines were extensively deployed worldwide and demonstrated significant efficacy in reducing severe disease and hospitalizations caused by COVID-19 [16,18]. In Hong Kong, a three-dose regimen of CoronaVac (SinoVac, 3 µg) achieved 97.9% protection against severe or fatal outcomes [44]. Similarly, in Chile, the same three-dose schedule reduced COVID-19-related hospitalizations by 78.8% [45]. In March 2020, during the early phase of the pandemic, our group successfully isolated the SARS-CoV-2 virus, subsequently designated as ERAGEM [36,37]. This isolate served as the foundation for the inactivated vaccine, initially named ERUCoV-VAC and later rebranded as TURKOVAC. TURKOVAC was developed using β-propiolactone-inactivated SARS-CoV-2 combined with aluminum hydroxide as an adjuvant. The vaccine demonstrated strong immunogenicity and a favorable safety profile in phase I, II, and III clinical trials. Phase III data established its non-inferiority to CoronaVac in reducing symptomatic COVID-19 cases and severe disease outcomes [18,19]. In 2021, TURKOVAC received emergency use authorization in Türkiye, marking a significant advancement in the global effort to combat the pandemic.

Despite the World Health Organization’s declaration of the end of the COVID-19 pandemic in May 2023, SARS-CoV-2 continues to pose challenges due to its rapid evolution, driven by high mutation rates, recombination events, and selective pressures [20,21]. VOCs, such as Delta and Omicron, have acquired mutations in the spike protein, particularly in the receptor-binding domain, enhancing infectivity, transmission efficiency, and immune evasion [35,46]. These dynamics have reduced the effectiveness of first-generation vaccines designed to target the ancestral strain [31,47]. Our study addressed these challenges by evaluating bivalent inactivated vaccines combining antigens from the ancestral ERAGEM strain with the Delta or Omicron (BA.5) variants. The findings from our study demonstrate that the bivalent inactivated vaccines effectively elicited strong immune responses and conferred complete protection after the challenge with the ancestral strain and the Delta and Omicron (BA.5) variants of SARS-CoV-2.

The variants selected for this study included the ancestral strain (ERAGEM), Delta, and Omicron (BA.5). The ancestral strain was chosen as it represents the original SARS-CoV-2 virus first identified in Wuhan, China, in December 2019 [48]. This strain has served as the foundation for most vaccine formulations developed to date, contributing to a well-documented and robust immune response in the global population [49]. Incorporating the ancestral strain in a bivalent vaccine is expected to strengthen the immunological response and provide a comprehensive base for broader protection [40]. The Delta variant was selected for its high transmissibility and its association with severe clinical outcomes, which have posed significant challenges to public health systems [30]. Delta has also acted as a genetic precursor to several subsequent variants, making its inclusion critical for mitigating severe disease outcomes and offering potential cross-protection against genetically related variants [31]. The Omicron (BA.5) variant was prioritized because it was responsible for one of the most widespread infection waves during the study period. While numerous Omicron sublineages have been circulating globally, the Omicron (BA.5) sublineage emerged as the dominant variant, making it a key target for vaccine development at that time [21]. Its widespread prevalence and its ability to escape immune defenses established by previous infections or vaccinations indicate the importance of incorporating Omicron (BA.5) into the vaccine formulation to enhance its relevance and efficacy against contemporary variants.

In this study, both vaccine formulations, ERAGEM + Delta and ERAGEM + Omicron (BA.5) achieved 100% survival in K18-hACE2 transgenic mice following challenges with their respective variants, demonstrating their efficacy in preventing severe disease outcomes (Figure 2). In the ERAGEM + Delta group, the endpoint antibody and neutralization titers against both the ERAGEM and Delta variants increased significantly after the prime-boost regimen, indicating a robust and broad immune response (Figure 4A and Figure 5A). Similarly, the ERAGEM + Omicron (BA.5) bivalent vaccine elicited substantial increases in GMTs for both ERAGEM and Omicron (BA.5), suggesting enhanced immunogenicity across these variants (Figure 4B and Figure 5B). Furthermore, no detectable viral titers were observed in the lungs of vaccinated mice challenged with either ERAGEM or the respective variant strains, confirming effective pulmonary protection (Figure 6). Significant reductions in the viral titers and RNA copy numbers in the nasal turbinates of the vaccinated groups compared to the controls further support the ability of bivalent vaccines to limit viral replication in the upper respiratory tract (Figure 7).

This study evaluates the efficacy of bivalent vaccine formulations, ERAGEM + Delta and ERAGEM + Omicron (BA.5), in providing protection against SARS-CoV-2 variants. When compared to the findings of a study conducted by our group in 2021 [37], which focused on a monovalent vaccine targeting the ancestral strain (ERAGEM), key similarities and potential advantages of the bivalent approach emerge. In terms of survival, both studies reported 100% survival in K18-hACE2 transgenic mice following challenges with the ancestral strain. Neutralizing antibody responses also provide an interesting point of comparison. In Pavel et al., the monovalent vaccine elicited a GMT of 157 against the ancestral strain following the booster dose. In our study, the ERAGEM + Omicron BA.5 bivalent vaccine achieved a GMT of 228.1 against the ancestral strain, while the ERAGEM + Delta vaccine elicited a comparable GMT of 157.1. While these results suggest that the bivalent vaccine formulations may generate immune responses at least equivalent to those for the monovalent vaccine against the ancestral strain, further studies are needed to confirm whether the inclusion of variant antigens enhances immunogenicity.

Although research on inactivated bivalent vaccines combining the ancestral (Wuhan) strain with Delta or Omicron variants is limited, recent studies have demonstrated the advantages of bivalent vaccines across different platforms. The bivalent mRNA-1273.214 booster demonstrated significantly higher neutralizing antibody titers against the Omicron sublineages, including the BA.4 and BA.5 variants, with the geometric mean ratios (GMRs) showing substantial improvement [50]. Similarly, the inactivated bivalent formulations in this study induced significant levels of neutralizing antibodies, with a noticeable increase observed after the booster dose. Booster administration plays a crucial role in enhancing the breadth and strength of cross-neutralization, as evidenced by the GMTs achieved against both the ERAGEM and variant-specific antigens. These findings are consistent with those of Liu et al., who demonstrated that inactivated vaccines can induce broadly neutralizing responses, even against immune-evasive sublineages. Similar to our findings, Wang et al. developed a bivalent RBD-based vaccine targeting the Wuhan-1 and Omicron BA.1 variants. This vaccine elicited durable antibody responses, including IgG and IgA, capable of neutralizing both the ancestral and variant strains and provided complete protection against nasal infection and disease in preclinical models [51,52]. Ying et al. also showed that a bivalent vaccine targeting the ancestral and Omicron BA.5 variants effectively protected against diverse variants, such as WA1/2020 D614G, BQ.1.1, and XBB.1.5, with no observed mortality in animal models [53]. Other studies emphasize the advantages of bivalent formulations in overcoming the antigenic variability of SARS-CoV-2. Li et al. demonstrated that bivalent mRNA vaccines incorporating the Delta and Omicron antigens elicited high neutralizing antibody titers against multiple strains, including variants not explicitly targeted by the vaccine [52]. Collectively, these findings demonstrate the critical role of bivalent vaccine formulations in overcoming the challenges posed by SARS-CoV-2 variants by generating robust, broad-spectrum immunity and offering protection across diverse viral strains.

While mRNA- and vector-based vaccines have received significant attention, our study highlights the distinct advantages of inactivated vaccine technology. This platform is built on a long-standing, reliable foundation with a strong safety record. Inactivated vaccines are compatible with existing production systems, making them highly accessible and practical for countries with limited resources. Unlike mRNA vaccines, which demand advanced manufacturing processes and ultra-cold storage, inactivated vaccines offer a more straightforward solution. Moreover, inactivated vaccines require only standard refrigeration (2–8 °C), which reduces the logistical hurdles often associated with mRNA vaccines that need storage at −20 °C or below. This characteristic makes inactivated vaccines an excellent option for large-scale distribution in regions with limited infrastructure or cold-chain capacity. Our research leverages these advantages by incorporating the regionally relevant ERAGEM SARS-CoV-2 isolate, ensuring its applicability to local epidemiological conditions. Paired with a comprehensive evaluation of immune responses against the Delta and Omicron (BA.5) variants, this approach addresses urgent public health needs while providing a viable framework for vaccine development in similar settings. By combining a targeted vaccine design with a globally scalable production platform, our work underscores its relevance and distinct contribution to the ongoing efforts to combat COVID-19.

One limitation of this study is the absence of monovalent vaccine groups, such as those targeting only the ancestral strain or specific variants such as Delta or Omicron (BA.5). Including such groups would have allowed for a direct comparison with the bivalent vaccine providing a clearer assessment of the advantages of bivalent formulations in eliciting broader immune responses and protection against a range of SARS-CoV-2 variants. Although this limitation does not diminish the significance of our findings, it represents an area for future research. Comparative studies incorporating both monovalent and multivalent vaccine groups could further validate the broader protection observed with bivalent vaccines. One of the other limitations of this study is the lack of direct evaluation of T-cell-mediated immune responses, which are increasingly recognized as critical in controlling SARS-CoV-2, particularly in mitigating severe disease outcomes. However, in our previous study using a monovalent inactivated vaccine based on the ancestral strain, we demonstrated that it primarily elicited humoral immunity while also inducing moderate cellular immune responses [37]. These findings suggest that the bivalent inactivated vaccines evaluated in this study may similarly stimulate cellular immunity, complementing the robust neutralizing antibody responses observed. Further investigation is needed to fully characterize the cellular immune mechanisms activated by these formulations, as such insights are essential for understanding their comprehensive protective potential.

## 5. Conclusions

In summary, this study demonstrates that inactivated bivalent vaccine formulations are capable of eliciting robust immune responses, reducing viral replication, and providing complete protection in preclinical models. These findings highlight their potential as a highly effective strategy for addressing the challenges posed by both ancestral and emerging SARS-CoV-2 variants, offering broad-spectrum immunity and significant protection against diverse viral strains. Future pandemics and outbreaks caused by rapidly mutating viruses are an inevitable global challenge. In this context, bivalent and multivalent vaccines play a pivotal role in ensuring preparedness for such events. By incorporating antigens from multiple strains, these vaccines can provide comprehensive protection against both known and emerging variants, reducing the health, social, and economic impacts of future pandemics.

## 6. Patents

Aykut Ozdarendeli, Shaikh Terkis Islam Pavel, Hazel Yetiskin, and Muhammet Ali Uygut are the named inventors on the patent applications covering inactivated COVID-19 vaccine development.

## Data Availability

The data that support the findings of this study are available upon request from the corresponding author.
